# Perspectives of radiographers on the emergence of artificial intelligence in diagnostic imaging in Saudi Arabia

**DOI:** 10.1186/s13244-022-01319-z

**Published:** 2022-11-22

**Authors:** Faten Mane Aldhafeeri

**Affiliations:** Department of Radiology, College of Applied Medical Sciences, University of Hafr Albatin, Hafr Al Batin, Saudi Arabia

**Keywords:** Artificial intelligence, Radiographers, Diagnostic imaging, Radiology

## Abstract

**Objectives:**

This study aimed to gain insight into radiographers’ views on the application of artificial intelligence (AI) in Saudi Arabia by conducting a qualitative investigation designed to provide recommendations to assist radiographic workforce improvement.

**Materials and methods:**

We conducted an online cross-sectional online survey of Saudi radiographers regarding perspectives on AI implementation, job security, workforce development, and ethics.

**Results:**

In total, 562 valid responses were received. Most respondents (90.6%) believed that AI was the direction of diagnostic imaging. Among the respondents, 88.5% stated that AI would improve the accuracy of diagnosis. Some challenges in implementing AI in Saudi Arabia include the high cost of equipment, inadequate knowledge, radiologists’ fear of losing employment, and concerns related to potential medical errors and cyber threats.

**Conclusion:**

Radiographers were generally positive about introducing AI to radiology departments. To integrate AI successfully into radiology departments, radiographers need training programs, transparent policies, and motivation.

## Key points

- Saudi radiographers have expressed a readiness to the use of artificial intelligence.
- Participants are concerned about their future employment prospects and lack of knowledge.
- Before AI implementation, intensive training programs and implementation must be performed.

## Introduction

Artificial intelligence (AI) is a subfield of computer science capable of performing tasks that typically require human intelligence. It is one of the fastest-growing subfields of informatics and computing, with the potential to significantly impact healthcare [1]. The use of AI in medical image production has led to changes in the role of radiographers, which benefits patients. To date, AI has focused on equipment and reducing radiation doses. There is no clear picture of how AI could be used in other areas [2].

Although AI-based image interpretation is perhaps the most well-studied task for improving the diagnosis of diseases in medical imaging, recent studies have focused on its application outside this scope with the goal of elucidating how to broadly enable imaging professionals to obtain ideal outcomes quickly [3]. Improved imaging workflows, image acquisition, pathology detection, research productivity, radiation dosage optimization, and high-standard medical care are just a few ways that AI tools are now being used in clinical settings [4, 5]. Furthermore, AI’s ability to accurately diagnose diseases has been reported to be comparable to that of humans [6].

Research has previously been conducted on radiographers’ attitudes regarding the application of AI and their readiness to incorporate AI into their clinical work [5, 7]. These studies focused on radiographers’ views on improving the process of implementing AI in medical imaging. Although these studies primarily used quantitative methodologies, they had methodological constraints that limited their ability to present various perspectives. At present, there is a shortage of studies using qualitative methods to examine the impact of AI on medical imaging.

Radiographers are crucial for integrating AI systems into medical imaging because they serve as an interface between technology and patients. Although some studies have examined how radiology workers feel about AI in Saudi Arabia [8, 9], we do not yet have a picture of their full perspective. This dearth is due to the fact that none of these studies focused on radiographers’ perspectives toward the integration of AI. Thus, this study aimed to gain insight into radiographers’ views on the application of AI in Saudi Arabia by conducting a qualitative investigation. We can expect radiographers to accept and prepare for AI based on the way that people generally form ideas about new technologies. Saudi Arabia tends to use AI techniques in other fields, such as health applications, and we anticipated that our participants would have good knowledge and perception of AI. The current study results will help in policy development and governance regarding AI integration.

## Materials and methods

### Study design

The local research ethics committee approved this study. This study employed a qualitative cross-sectional survey design using self-administered questionnaire adapted from a previously published study [10]. The study used a non-probability convenience sampling technique. The target group in this study was radiographers from all regions around Saudi Arabia. According to the statistical yearbook issued by Saudi Ministry of Health, there are 7719 registered radiographers with the Saudi Commission for Health Specialties (the national regulatory body for health practitioners in Saudi Arabia). G*Power version 3.1.9.7 was used to determine the minimum sample size for the investigation (*n* = 368). Between November 2021 and May 2022, data for this study were collected via an electronic questionnaire created using Google Forms (Google, Mountain View, CA, USA). The link was distributed throughout Saudi Arabia via email, WhatsApp groups, and Twitter with frequent reminders to maximize response. The study is a multicenter, nationwide with prospective data collection. The sample consists of radiographers with similar cultural and linguistic backgrounds from each of Saudi Arabia’s thirteen geographical areas. The hospitals/health centers included public, private, and University medical hospitals that provide medical services across the 13 Saudi geographical regions. Radiographers who are working in administrative positions were excluded from the study. In order to maintain privacy, all responses were recorded anonymously and then encrypted before being transferred to a computer. Participants were provided a description of the aim, risk, reward, questionnaire duration, and nature of AI. In addition, participants could withdraw from the study with no consequence at any time. They were also notified that the questionnaire was restricted to radiographers who worked in Saudi Arabia and agreed to participate. On the first page of the questionnaire, each radiographer was asked to electronically consent to their participation in order to access the survey. The questionnaire included questions on demographics, general opinions and viewpoints on AI, thoughts on how AI should be deployed in Saudi Arabia, job security, workforce development and other aspects of the future of medical imaging, and the ethics surrounding the integration of AI into clinical practice. A pilot study was done using a population sample, and a 10-min completion time was anticipated.

### Statistical analysis

We used SPSS version 24 (IBM Corp., Armonk, New York, USA) for data collection, classification, and processing. We used a Likert scale (strongly agree = 5, agree = 4, not sure = 3, disagree = 2, and strongly disagree = 1) to assess responses to rating questions. “Strongly agreed” and “agreed” responses were grouped as an “agreement response,” whereas “strongly disagree” and “disagree” responses were grouped as a “disagreement response.” The quantitative variables were expressed as percentages, mean, and standard deviations. Spearman’s correlation was used to analyze the correlation between radiographers’ attitudes toward AI and demographic factors. A two-tailed value of 0.05 was applied to all statistical significance tests.

## Results

Of the 562 responses received, 64.7% (*n* = 364) were from men. Participants’ mean age (± standard deviation) was 31.6 ± 6.6 years. Table 1 presents the respondents’ demographic characteristics. None of the respondents aged > 50; in fact, this might be due to exclusion of any radiographer who is not practicing the profession or working in administrative position. It is worth mentioning that most of Saudi radiographers who worked for many years in medical imaging departments move to administrative work. And another explanation, even if there are practicing radiographers > 50 years despite their few number, perhaps they did not participate in the survey. Table 2 reveals that most respondents (90.7%, *n* = 510) viewed AI technology as being the future of diagnostic imaging. Similarly, a large majority of respondents (*n* = 412, 73.3%) indicated that AI would positively affect medical imaging practice. Others (*n* = 368, 65.4%) indicated that AI decreases radiation exposure levels while preserving optimal image quality (Table 3). The majority of respondents (*n* = 448, 79.7%) were concerned about potential machine errors related to using AI-integrated equipment in radiography practice, as presented in Table 4. Table 5 includes different responses from respondents regarding aspects that can influence AI implementation and associated decision-making in medical imaging. High installation costs (*n* = 478, 85.0%), lack of expertise (*n* = 432, 76.8%), and perceived cyber threats (*n* = 370, 65.8%) were identified as obstacles to the implementation of AI in Saudi Arabia.

**Table 1:** Demographic distribution of participants

Variable	N (%)
Age	
20–29	240 (43%)
30–39	292 (52%)
40–49	28 (5%)
> 50	0 (0%)
Sex	
Male	428 (76%
Female	134 (24%)
Years of Experience	
0–5 years	140 (25%)
6–10 years	138 (24.5%)
11–15 years	238 (42%)
> 15 years	46 (8.5)
Educational Level	
Diploma	174 (31%)
Bachelor’s degree (BSc)	252 (45%)
Master’s degree	124 (22%)
Doctor of Philosophy (PhD)	12 (2%)
Modality used by participant	
General X-ray	202(36%)
CT	124 (22%)
MRI	96 (17%)
Fluoroscopy	62 (11%)
Mammography	22 (4%)
Ultrasound	44 (8%)
Other modalities	12 (2%)
Work setting	
Governmental	404 (72%)
Private	40 (7%)
Military	44 (8%)
Quasi-government	74 (13%)

**Table 2:** General thoughts and views of respondents toward clinical use of AI in diagnostic imaging

Item	Agreement	Neutral	Disagreement	M (SD)
AI is a new trend in diagnostic imaging that I am aware of	404 (72%)	100 (17.8%)	58 (10.2%)	4.1 (0.7)
Emergence of AI in the Saudi radiography industry	360 (64%)	40 (7%)	162 (29%)	4.5 (0.65)
Concerns exist with the adoption of AI into diagnostic imaging	422 (75%)	28 (5%)	112 (20%)	3.5 (0.98)
The implementation of AI in diagnostic imaging excites me	483 (86%)	55 (9.7%)	24 (4.3%)	4.1 (1.1)
I think the majority of patients would be enthusiastic about the application of AI in their healthcare	415 (73.8%)	123 (22%)	24 (4.2%)	3.3 (0.87)
I believe AI to be the future of radiology	510 (90.6%)	16 (3%)	36 (6.4%)	4.3 (0.85)

**Table 3:** Respondents’ thoughts on the potential positive effects of AI in diagnostic imaging

Item	Agreement	Neutral	Disagreement	M(SD)
AI would have a beneficial overall influence on diagnostic imaging	412 (73.3%)	72 (12.7%)	78 (14%)	3.7 (0.8)
The majority of my patients would be delighted by AI in healthcare	454 (80.7%)	72 (12.8%)	36 (6.5%)	4.3 (0.9)
AI might assist minimize radiation exposure levels in diagnostic imaging while retaining optimum image quality	368 (65.4%)	62 (11%)	132 (23.6%)	4.2 (0.7)
AI would be a helpful tool in my profession as a radiographer	410 (72.8%)	102 (18.3%)	50 (8.9%)	4.4 (0.8)
In areas where radiologists are unreachable, AI might improve patient care access	426 (75.8%)	82 (14.6%)	60 (9.6%)	4.2 (0.8)
The use of AI in diagnostic imaging facilitates increased research output in radiology	372 (66.3%)	134 (24%)	36 (9.7%)	3.9 (1.1)
AI would significantly improve the accuracy of both illness detection and diagnosis	498 (88.5%)	44 (7.8%)	20 (3.7)	4.3 (0.9)
AI would improve education in medical imaging	464 (82.6%)	70 (12.4%)	28 (5%)	3.7 (1.2)
Patients’ diagnostic outcomes might benefit from AI-aided decision-making	524 (93.4%)	30 (5.4%)	6 (1.2%)	4.1 (1.1)
There will be a modification in the responsibilities of radiographers because of the use of AI	376 (67%)	68 (12%)	118 (21%)	3.9 (1.1)

**Table 4:** Respondents’ thoughts on the potential negative effects of AI in diagnostic imaging

Item	Agreement	Neutral	Disagreement	M (SD)
The adoption of AI would restrict the radiographer’s job in the department	318 (56.6%)	48 (8.6%)	196 (34.8%)	3.7 (1.3)
The implementation of AI in diagnostic image interpretation will adversely impact the majority of radiologists	274 (48.7%)	130 (23.3%)	316 (28%)	3.8 (1.1)
I’m worried that (AI) will eventually replace me in my career path	238 (42.6%)	106 (18.7%)	218 (38.7%)	4.2 (0.9)
I feel that AI, as an assisting tool, may reduce my income in the future	212 (37.8%)	94 (16.8%)	276 (45.4%)	4.2 (0.8)
I am aware of the probability of machine mistakes in the radiology unit due to AI-induced equipment	448 (79.8%)	38 (6.6%)	76 (13.6%)	4.3 (0.8)
By storing personal information with health data, AI may violate patients’ rights to privacy and confidentiality	216 (38.6%)	82 (14.4%)	264 (47%)	3.7 (1.2)
It’s possible that the usage of AI techniques may lead to the unauthorized commercial exploitation of patient data	160 (28.7%)	42 (7.3%)	360 (64%)	3.8 (1.3)

**Table 5:** Perspectives on the determinants that affect AI deployment and decision-making in medical imaging

Item	Agreement	Neutral	Disagreement	M (SD)
AI deployment in Saudi Arabia will be constrained by high installation costs	478 (85%)	32 (5.6%)	52 (9.4%)	4.2 (0.9)
I recognize that a lack of understanding about the advent of AI technology is a key impediment to AI deployment	432 (93%)	6 (1%)	34 (6%)	4.2 (0.8)
The application of AI is vulnerable to cyber danger	370 (65.7%)	60 (10.7%)	132 (23.6%)	3.9 (1.1)
In the absence of effective cyber security measures, cybercriminals may control AI	400 (71%)	28 (5%)	134 (24%)	4.2 (0.7)
AI algorithmic and diagnostic decisions should be shared equally	256 (45.6%)	94 (16.6%)	212 (37.8%)	3.7 (1.2)
The diagnostic decision-making process should be conducted by an AI system	130 (23%)	118 (21%)	314 (56%)	3.8 (1.1)
Who is responsible for a diagnostic error caused by AI-tool software?	86 (15.6%) The radiographer in charge 358 (63.6%) The machine manufacturers 40 (7%) The referring radiologist 78 (13.8%) Others, for example, AI administrators, handlers, and health facility			3.5 (1.2) 4.1 (0.9) 3.3 (1.3) 3.2 (1.1)

There were no statistically significant differences in sex in terms of attitudes and perspectives toward AI (*p* = 0.076), as well as the positive and negative impact of AI (*p* = 0.27 and *p* = 0.085, respectively). Additionally, the results did not reveal a statistically significant difference between years of experience and perspectives and attitudes toward AI (*p* = 0.47) and its positive and negative impact (*p* = 0.86 and *p* = 0.37, respectively). Respondents’ educational level was positively correlated with the general attitudinal perspective (*p* = 0.03) and AI’s positive and negative impact (*p* = 0.01 and *p* = 0.04, respectively). A post hoc multiple comparisons revealed a statistically significant difference between groups for respondents who hold PhD qualification (*p* = 0.034) and believe that AI is the future of radiology. Results of the post hoc test also revealed a significant difference between groups for respondents who had PhD (*p* = 0.04) and believe that AI might assist minimize radiation exposure levels in medical imaging. A post hoc test revealed no difference between groups in terms of the imaging modality used by respondent.

## Discussion

AI may dramatically enhance the performance of health practitioners. In radiology, the transition to AI may help reduce radiographers’ workload and improve image acquisition and quality assurance. However, there is minimal research on how radiology workers in Saudi Arabia might interpret such changes. Saudi Arabia has used AI in various industries, particularly in the health sector, where there are numerous applications that chronicle the population’s health status, such as vaccines and COVID-19 infections in the pandemic. The Saudi Arabian government has established a national center for AI because it believes in its usefulness in various disciplines.

However, this technique has not yet been used in radiology. Radiology departments are undergoing a tremendous technological revolution that will markedly impact the profession [2, 11]. Before adopting this technique, it is crucial to assess radiographers’ knowledge and attitudes about AI. To the best of our knowledge, this is the first study to comprehensively assess the perspectives of radiographers from across Saudi Arabia regarding the integration of AI in radiology departments.

This survey aimed to assess Saudi radiographers’ perspectives on the implementation of AI in medical imaging. The majority of respondents (73.3%) knew that AI is an emerging trend in medical imaging, while 90.6% viewed it as the discipline’s future. This finding is similar to that of Botwe et al. [10] who reported that most participants (86.1%) agreed that AI would be the future of medical imaging. Abuzaid et al. [7] also reported that most radiographers in the Middle East and India believe that AI plays an important role in radiology. Alelyani et al. [9] also said that 61.2% of the radiological community in Saudi Arabia was aware of AI and its role in radiology. Similar excitement toward AI implementation in clinical diagnosis has also been reported by Sarwar et al. [12] who predicted a complete integration of AI within the next five years.

Regarding the positive impact of AI, most participants (72.8%) felt that it might be a helpful tool to facilitate their jobs as radiographers. This outcome will increase the number of patients examined by the MRI technician. Most respondents (65.4%) had a favorable opinion regarding the role of AI for dose optimization and image quality. Most radiographers (66.3%) felt that implementing AI in radiology departments would give them the ability to conduct research and be productive. Current findings align with those of previously published studies [3, 13]. Most respondents (93.4%) believed that the implementation of AI in radiology would improve decision-making regarding patients’ diagnostic results. The ability of AI-based decision support systems to deliver accurate diagnostic findings by triaging and flagging aberrant patient images has been reported [4, 6]. These insights are reassuring, because the issues discussed are crucial to radiography practice.

The emergence of AI in radiology raises questions about its potential impact on radiographer employment. More than half the respondents reported that the integration of AI would limit their work in the units, and a large proportion were concerned about displacement from their jobs. In addition, they even believed that radiologists’ jobs are affected by the introduction of AI in diagnostic image interpretation. Similarly, previous studies [8, 14] have found that radiologists have some concerns regarding their future job security due to the growing trends in AI technologies. The decrease in image acquisition time in MRI is an advantage of AI implementation in radiology departments. Hence, respondents seemed to agree that AI would facilitate the radiographer’s job. However, this will increase the number of daily patients examined by radiographers and thus increase the workload. This is similar to a study conducted by Botwe et al. [15] who found that radiographers agreed that the implementation of AI in medical imaging departments would “ease” their work. This perception might be influenced by arguments made in the literature that AI is expected to speed up tasks. In fact, there is some debate over whether AI would increase or decrease workload in radiology departments [16]. Many medical students do not consider radiology a future career option due to AI’s integration [17]. Although there is widespread concern that AI will replace human jobs [18], there seems to be no evidence to support this hypothesis [4]. A recent study showed that AI may be misunderstood, which may explain this belief [5].

Understanding the function of AI in medical imaging may be improved by better communication across departments and clear guidelines and policies. There was also a proportion (37.8%) of those who felt that the integration of AI would reduce their salary. It is also important to emphasize that AI cannot take the place of humans in terms of, for example, patient positioning or communication. The majority of respondents (79.8%) expressed concerns that the use of AI in radiology was associated with machine errors. Ophthalmologists and radiologists have also reported similar concerns [8, 19]. Some respondents (28.7%) were concerned about using AI tools, as this could lead to illegal utilization of patient data for inappropriate commercial purposes. This is because AI-powered devices require patient data for quality and system training [20]. However, humans who employ AI will be held responsible for avoiding these faults because AI does not integrate ethical ideas such as equality [21]. This highlights the urgent need for AI governance regulations before its deployment in Saudi Arabia.

Of note, radiographers’ perspectives on the impact of AI were not correlated with age or years of experience but rather with educational level. This might be explained by the fact that curricula for bachelor’s degrees and above contain courses on computers and programming, while the diploma curricula, although discontinued long ago, lacked computing courses. This implies that radiographers should be trained according to their educational level. However, these findings are not consistent with previous study results [10, 15]. The geographical and socioeconomic backgrounds of the current and other respondents could explain, at least in part, the differences observed in this research.

With regard to potential study limitations, we recognize that the possibility for bias in qualitative research studies is debatable. In qualitative research, bias may result from the way the question is phrased, the method by which the participants reply, and the researchers’ expectations. We did not include in our questionnaire open-ended questions that would enable participants to elaborate on their specific worries and challenges with AI, which might be considered as a limitation of this study. Another limitation of this study is that it is multicenter study in only one country. Further studies should address the international perspectives from radiographers from multiple countries.

Overall, these findings imply that radiographers working in Saudi Arabia are optimistic about implementing AI in medical imaging. However, apprehensions regarding job security are a major concern for the integration of AI in medical imaging. As with previous transformational and revolutionary technologies, the deployment of AI in medical imaging in Saudi Arabia may be difficult. Lack of expertise, regulatory laws, and support systems have been cited as significant obstacles to the effective adoption of AI, which stakeholders should address. The results indicated that radiographers struggled to obtain AI-related education and training. This difficulty is exacerbated because the radiographers have noted a shortage of post-qualification education courses. This study provides novel insights and suggestions to enhance the training of the Saudi radiography workforce and others in similar resource-limited environments to offer quality care utilizing AI-integrated imaging modalities.

## Data Availability

The datasets used and/or analyzed during the current study are available from the corresponding author on reasonable request. All authors read and approved the final manuscript.
